# Ultrasonographic Findings of Renal Cell Carcinomas Associated with Xp11.2 Translocation/TFE3 Gene Fusion

**DOI:** 10.1155/2017/2958357

**Published:** 2017-11-23

**Authors:** Wenwu Ling, Xuelei Ma, Yan Luo, Linyan Chen, Huiyao Wang, Xiaoling Wang, Ni Chen, Hao Zeng, Yongzhong Li, Diming Cai

**Affiliations:** ^1^Department of Ultrasound, West China Hospital, Sichuan University, Chengdu, China; ^2^State Key Laboratory of Biotherapy and Cancer Center, West China Hospital, Sichuan University and Collaborative Innovation Center, Chengdu, China; ^3^Department of Psychiatry, West China Hospital, Sichuan University, Chengdu, China; ^4^Department of Operations Management, West China Hospital, Sichuan University, Chengdu, China; ^5^Department of Pathology, West China Hospital, Sichuan University, Chengdu, China; ^6^Department of Urology, West China Hospital, Sichuan University, Chengdu, China

## Abstract

**Objective:**

This study was to investigate the features of renal carcinomas associated with Xp11.2 translocations/TFE3 gene fusions (Xp11.2-RCC) on conventional ultrasound (US) and contrast-enhanced ultrasound (CEUS).

**Methods:**

US and CEUS features of twenty-two cases with histopathologically proven Xp11.2-RCC were retrospectively reviewed.

**Results:**

22 patients (11 males, 11 females) were included in this study, with a mean age of 28.3 ± 20.4 years. Eight tumors (36.3%, 8/22) were in left kidney, and 14 tumors (63.7%, 14/22) were in right kidney. All tumors (100%, 22/22) were mixed echogenicity type. 13 tumors (59.1%, 13/22) presented small dotted calcifications. The boundary of 14 tumors (63.6%, 14/22) was sharp and the other 8 tumors' (36.4%, 8/22) boundary was blurry. By CEUS, in early phase, the solid element of all tumors showed obvious enhancement. In delayed phase, 13 tumors showed hypoenhancement, seven tumors showed isoenhancement, and 2 tumors showed hyperenhancement. There were irregular nonenhancement areas in all tumors inside.

**Conclusions:**

By US and CEUS, when children and adolescents were found to have hyperechoic mixed tumor in kidney with sharp margin and calcification, and the tumors showed obvious enhancement and hypoenhancement with irregular nonenhancement areas in the tumor in early phase and delayed phase, respectively, Xp11.2-RCC should be suspected.

## 1. Introduction

Renal cell carcinoma associated with Xp11.2 translocation/TFE 3 gene fusion (Xp11.2-RCC) is a rare subtype of RCC that is now accepted as a distinct entity according to the 2016 World Health Organization renal tumor classification [1]. In the clinical works, cases of Xp11.2-RCC were found by postoperation of pathology, confirmed now and then. In the literatures, the medical imaging of Xp11.2-RCC was converged by computer tomography (CT) or magnetic resonance imaging (MRI) [2–5]. Xp11.2-RCC is typically presented as asymptomatic, painless renal mass and is often identified accidentally by abdominal imaging [6]. Ultrasound is the most widely used in abdominal examination because it is cheap and convenient with no radiation exposure. But few cases had been diagnosed by US and CEUS in the literatures. Are there any features of Xp11.2-RCC by US and CEUS? We designed this retrospective study to answer the question.

## 2. Materials and Methods

### 2.1. Patient Data

This study was conducted in accordance with the declaration of Helsinki. This study was conducted with approval from the Ethics Committee of West China Hospital, Sichuan University. We retrospectively reviewed the results of US and CEUS examination of 22 patients (11 males, 11 females, mean age: 28.3 ± 20.4 years, range: 6 to 63 years) with 22 tumors of Xp11.2-RCC who were admitted to our hospital between January 2009 and January 2017. And all cases were confirmed by pathology postoperatively. The tumors were diagnosed by pathology not only on morphology itself but also on immunophenotype and molecular genetics findings (fluorescence in situ hybridization, FISH; reverse transcriptase polymerase chain reaction, RT-PCR; or next-generation sequencing, NGS).

### 2.2. US Examination

US and CEUS were performed with a Philips IU22 scanner (Philips Medical Solutions, Mountain View, CA, USA) with a 1–5-MHz convex transducer or LOGIQ E9 (GE Healthcare, Milwaukee, WI, United States) ultrasound system with a C2–5 MHz probe. The US systems were equipped with harmonic contrast pulse sequencing apparatus. The contrast agent used was SonoVue (BraccoSpa, Milan, Italy) and the suspension contained stabilized sulfur hexafluoride microbubbles. The examinations were performed by two sonologists (Cai DM, Ling WW) who had >5 years of experience in renal CEUS. After conventional US, CEUS was performed. Then, CEUS was started at a low mechanical index (PHILIPS MI: 0.06; GE MI: 0.12). SonoVue suspension (2.4 mL) was administered as a bolus injection through the antecubital vein, followed by a flush with 5 mL saline solution. Each study involved active monitoring of the lesion of interest and surrounding areas in the early phase (range, 0 s to 30 s), late phase (range, 60 s to 120 s), and delayed phase (>120 s).

### 2.3. Image Analysis

The location, size, shape, boundary, and inner echogenicity of the lesions were observed and recorded by US. The origins of the tumors were evaluated whether they possibly originated from the renal cortex or renal medullary tissue or are indistinct. By CDFI and PW mode, the blood flow was observed and recorded. The renal veins of affected side were evaluated whether there is embolism, even with inferior vena cava (IVC). The enhancement pattern and enhancement level in different phases of CEUS imaging were reviewed. The degree of enhancement was divided into nonenhancement, hypoenhancement, isoenhancement, and hyperenhancement, according to the enhancement level of the lesion compared with that of the surrounding normal renal parenchyma. Contrast enhancement patterns were recorded by two physicians (Cai DM, Ling WW).

## 3. Results

### 3.1. US Findings

**Figure 1:**
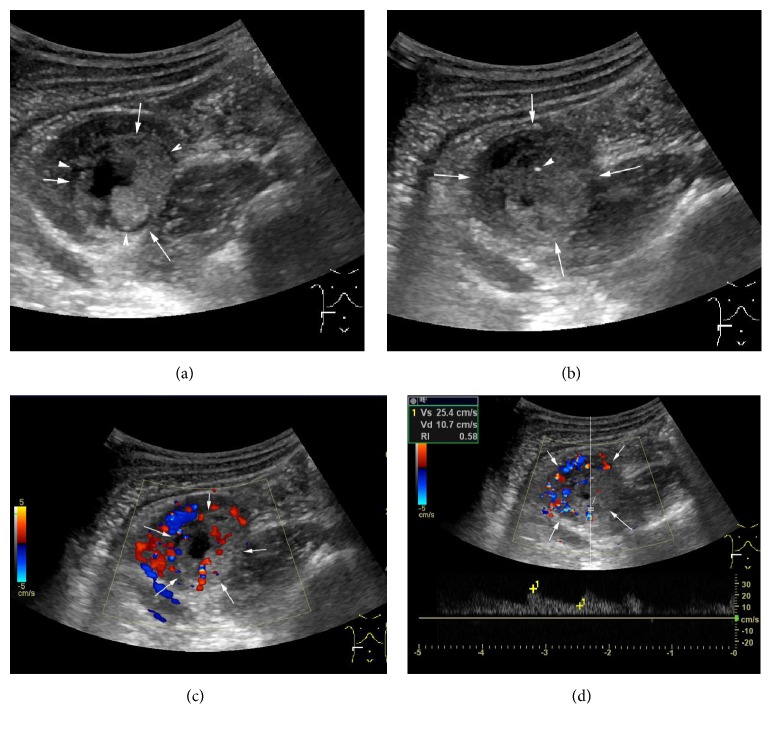
Case 1: male, 12 years old; a hyperechoic mixed mass was found in his right renal of lower part in the renal medulla. (a) Cyst-solid mass with size of 3.2 × 2.1 cm was found (arrow) and an annular hypoechoic halo sign surrounding the tumor was displayed (arrow head). (b) Spot calcification (arrow head) was found inside the tumor (arrow). (c) By CDFI mode, the solid component was found by multibranch threadiness color Doppler signal. (d) By PW mode, the solid component was detected by low blood flow resistance indexes (RI = 0.58).

In total, all tumors were found by US. Eight tumors (36.3%, 8/22) were in left kidney and 14 tumors (63.7%, 14/22) were in the right. The range size of the tumors was 2.7 × 2.8 cm–13 × 8 cm. 14 tumors (63.7%, 14/22) were of solid-cyst mixed type, 5 tumors (22.7%, 5/22) were of multilocular cysts, and 3 tumors (13.6%, 3/22) were solid. 13 tumors (59%, 13/22) displayed hyperechogenicity, 6 tumors (18.2%, 6/22) were hypoechoic, and 5 tumors (22.7%, 5/22) were multilocular cystic. 13 tumors (59.1%, 13/22) presented small dotted calcifications. The boundary of 14 tumors (63.6%, 14/22) was sharp and the other 8 tumors' (36.4%, 8/22) boundary was blurry. 13 tumors (59.1%, 13/22) had close relations with renal medulla and the others (40.9%, 9/22) were indistinctive huge tumors of which the origins could not be confirmed. That was to say that renal cortex and renal medulla were all involved in the tumors (Figure 1). Thrombosis was found in the left renal vein in only one case (4.5%, 1/22).

### 3.2. CEUS Findings

**Figure 2:**
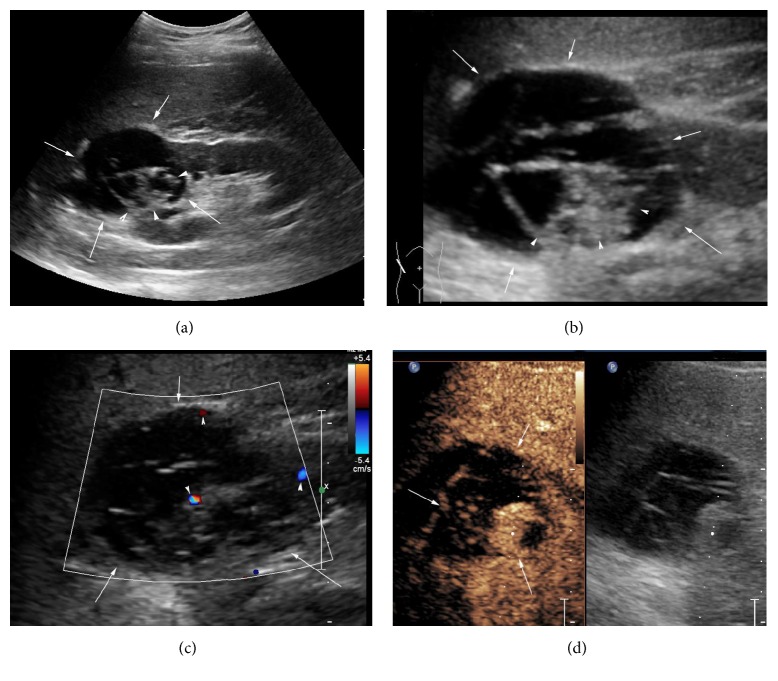
Case 2: male, 48 years old; a multilocular cystic was found in his right renal upper pole. (a) An multilocular cystic with size of 6.0 × 5.1 × 5.8 cm was found (arrow) and irregular thick walls inside the tumor was displayed (arrow head). (b) By partial enlarged view, the multilocular cystic was displayed in detail (arrow) and the irregular thick wall (arrow head). (c) By color Doppler flow imaging (CDFI) mode, the cystic thick walls (arrow), the subcapsular of tumor, and the nodule inside tumor were detected by spot color Doppler signal. (d) By CEUS, the nodule inside the tumor showed hyperenhancement and the thick walls showed isoenhancement in the early phase (arrow).

22 tumors were all detected on CEUS. In early phase (range, 0 s to 30 s), the solid element of the tumors showed obvious enhancement compared to the renal parenchyma. In the late phase (range, 60 s to 120 s), 7 tumors of solid element showed hyperenhancement, 8 tumors showed isoenhancement, and 7 tumors showed hypoenhancement. In delayed phase (>120 s), 13 tumors showed hypoenhancement, 7 tumors showed isoenhancement, and 2 tumors showed hyperenhancement. Irregular areas with no-enhancement and the washout of contrast agents were found in each tumor (Figure 2).

### 3.3. Pathological Findings

**Figure 3:**
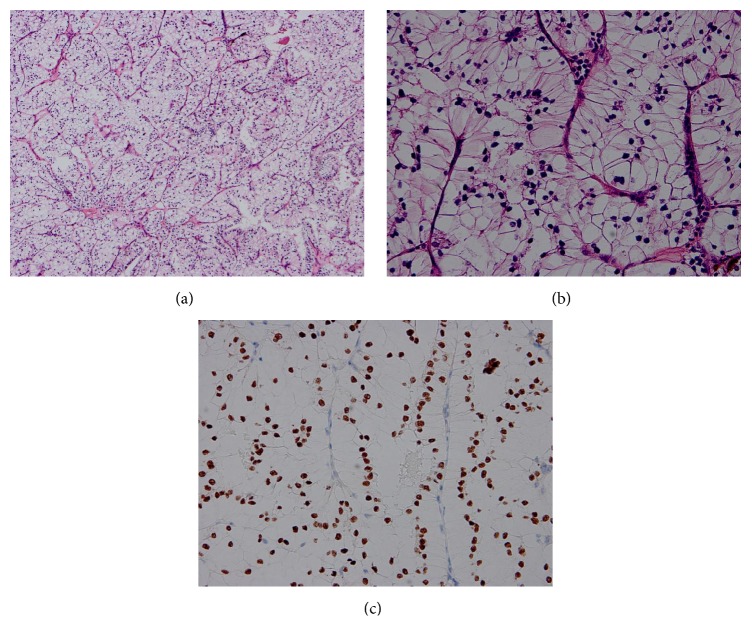
Pathological findings. (a) The tumor cells were polygonal of a papillary carcinoma with clear cells and cells with granular eosinophilic cytoplasm (a) (HE staining, 100x magnification) and (b) (HE staining, 400x magnification). (c) The tumor cells in the kidney were visualized by immunohistochemistry staining for TFE3 which revealed positive staining of the nuclei.

The histopathologic appearance was that the tumor cells were polygonal of a papillary carcinoma with clear cells and cells with granular eosinophilic cytoplasm. These cells displayed nuclear immunoreactivity for TFE3 protein in all 22 cases, which supported the diagnosis of Xp11.2-RCC (Figure 3). To avoid the misdiagnosis, FISH assays were implemented in the tumors. The signals of tumors were split by FISH assays, and all tumors showed positive results.

## 4. Discussion

Xp11.2-RCC is a rare subtype of RCC that usually affects children and adolescents in reports [1, 7–9]. In our study, 14 patients (63.6%) were younger than 30 years old. It was consistent with previous reports [10]. In our study, the ratio of males to females was 1 : 1 (11 : 11), consistent with the previous report of no gender difference [11], different from the male predominance reported by Dang et al. [12].

The clinical symptoms of these carcinomas are not clear yet. In our study, the symptoms in 31.8% (7/12) of the patients were nonspecific, and the other 68.2% (15/22) were detected incidentally. Adult-onset Xp11.2-RCC, unlike those with onset during childhood, demonstrated more aggressive clinical courses [13–15]. In our study, 11 cases (50%) were over 20 years old. One adult-onset patient was 61 years old with left renal vein thrombus. A 15-year-old patient, loss of consciousness, was admitted to our emergency department 1 month after nephrectomy. This patient was diagnosed with metastatic renal tumor of spinal canal by CT. And the patient refused further treatment and his prognosis was misadventure.

To date, the imaging features of Xp11.2-RCC by ultrasonography have not been reported and few studies reported its CT or MRI features. Because these tumor cases are very few around the world, we retrospectively analyzed the imaging features by US of Xp11.2-RCC using a relatively large sample. These merits helped to reveal the general imaging features. (1) The tumor may be originated from the proximal or distal nephron. In our study, 13 tumors (59.1%, 13/22) (diameter ⩽ 5 cm) were found in the renal proximal. When it is large, the neoplasm invaded the surrounding tissue and bulged with kidney contours. And this imaging feature was different from the clear cell renal cell carcinoma (CCRCC) which is the most common malignancy in kidney. It is well known that CCRCC originated from the renal cortex tissue. And this character may be a criterion in differential diagnosis between the two tumors by US. (2) Xp11.2-RCC may be cystic-solid mixed mass, with irregular solid and liquid interphase component in the tumor. And in our study, 14 tumors (63.7%, 14/22) were of solid-cyst mixed type, 5 tumors (22.7%, 5/22) were solid, and 3 tumors (13.6%, 3/22) were multilocular cysts where some nodules were found at the internal face of cysts by US. This feature was related to the tumor pathological change, with hemorrhage, necrosis, or cystic changes inside the tumor [16, 17]. (3) Calcification may be found in the internal tumor of Xp11.2-RCC. In our study, punctate calcification was found in 13 internal tumors (59.1%, 13/22). The calcifications were confirmed by pathology. In them, two tumors were spot calcification and the others were irregular. Calcification cannot diagnose the rare malignancy directly, but in the type of RCC, calcification could be found [3]. (4) The tumors may be detected with rich color Doppler signals in the solid component of tumor by CDFI mode. The features of this may be connected with tumor's pathological basis. In previous report, the tissue of solid component in the tumor was found with plenty of blood capillary and arteriovenous shunting [3]. (5) The margins of tumors may be sharp. In our study, 14 (63.6%, 14/22) tumors' margin was sharp by US. The other margin was blurry. By CEUS mode, 17 (72.3%, 17/22) tumors' margin was sharp. According to the operation records, 17 tumors (72.3%, 17/22) have pseudocapsule which made the resection easy and whole. Because of the pseudocapsule, the tumor's margin was sharp. This was consistent with the literature reported previously in that the pseudocapsules were found in tumor with fibrous connective tissue by pathology [18].

This study had several limitations. Because Xp11.2-RCC is an uncommon RCC subtype, small sample size will lead to selection bias.

As concluded by US, when children and adolescents were found to have hyperechoic mixed tumor in kidney, with sharp margin, close relation with renal medulla, rich CDFI signal, calcification, and Xp11.2-RCC should be suspected. By CEUS, in the early phase when the tumors showed obvious enhancement and in delayed phase when tumors showed hypoenhancement with irregular areas inside the tumor with nonenhancement, Xp11.2-RCC should be suspected.
